# Age-Dependent Reference Values for hs-Troponin T and NT-proBNP and Determining Factors in a Cohort of Healthy Children (The LIFE Child Study)

**DOI:** 10.1007/s00246-022-02827-x

**Published:** 2022-03-12

**Authors:** Alexandra Kiess, Jessica Green, Anja Willenberg, Uta Ceglarek, Ingo Dähnert, Anne Jurkutat, Antje Körner, Andreas Hiemisch, Wieland Kiess, Mandy Vogel

**Affiliations:** 1 Heart Center Leipzig, Department of Pediatric Cardiology, Faculty of Medicine, University of Leipzig, Strümpellstraße 39, 04289 Leipzig, Germany; 2 Pediatric Intensive Care Unit, The Royal Children’s Hospital, Flemington Road, Parkville, VIC 3052 Australia; 3 Institute of Laboratory Medicine, Clinical Chemistry, and Molecular Diagnostics (ILM), University of Leipzig, Liebigstrasse 27, 04103 Leipzig, Germany; 4 LIFE Leipzig Research Center for Civilization Diseases, University of Leipzig, Philipp-Rosenthal-Strasse 27, 04103 Leipzig, Germany; 5 Department of Women and Child Health, Hospital for Children and Adolescents and Center for Pediatric Research (CPL), University of Leipzig, Liebigstrasse 20a, 04103 Leipzig, Germany

**Keywords:** Cardiac biomarkers, Pediatric percentiles, Troponin T, NT-ProBNP

## Abstract

This study aimed to provide reliable pediatric reference values for N-terminal pro-brain natriuretic peptide (NT-proBNP) and high-sensitive Troponin T (hsTnT) obtained from a population of well children and investigate for associations with sex, pubertal status, body mass index (BMI), and serum lipid levels. We analyzed hsTnT and NT-proBNP values obtained from 4826 samples provided by 2522 children aged 0.25–18 years participating in a prospective longitudinal population-based cohort study, “LIFE child” in Leipzig, Germany (Poulain et al., Eur J Epidemiol 32:145–158, 2017). NT-proBNP values decreased throughout childhood from values over 400 ng/L at 3 months to 138 ng/L in females and 65 ng/L in males by 18 years of age. Values dropped rapidly with advancing pubertal stage. We found a strong association between lower NT-proBNP values and higher BMI or elevated serum lipids, the latter effect being more pronounced in males. For hsTnT levels, approximately half of the measurements were below the detection limit. However, 76% of those aged 3 months and 21% of those aged 6 months had values exceeding the adult cut-off limit. Females had slightly higher levels in the first 2 years of life but this was reversed during puberty. In males, there was an upward trend from pubertal stage 2 onward. We identified a positive association between hsTnT and BMI but a negative association with low-density lipoprotein (LDL) cholesterol and triglyceride levels in boys but not in girls. Based on a large number of healthy children, we have established reliable reference values for NT-proBNP and hsTnT for use in everyday clinical practice. We have also identified important associations between certain metabolic and cardiac markers.

**Clinical Trial Registration** ClinicalTrial.gov (NCT02550236).

## Background

N-terminal pro-brain natriuretic peptide (NT-proBNP) and high-sensitive Troponin T (hsTnT) are well-established parameters for diagnosing acute and chronic cardiac stress in adults. They are commonly applied in pediatric patients, despite lacking reliable reference values for this population. For example, both markers were used recently in the biochemical diagnosis of multisystem inflammatory syndrome in children (MIS-C) utilizing adult cut-off values [2].

Previous studies investigating NT-proBNP and hsTnT in children have limitations, conducted in small study populations, not encompassing all pediatric age groups, or confounded by being collected during presentation for inter-concurrent illness or surgery [3–6]. In adults, population-based studies showed consistently negative associations between NT-proBNP and metabolic markers, such as BMI and lipid levels [7–9], whereas the limited data available from pediatric cohorts have not demonstrated this correlation [10, 11]. These divergent findings could be due to different metabolic mechanisms in childhood or secondary to a sampling error from small study participant numbers. The LIFE Child cohort, a population-based cohort from Leipzig, Germany, provides both laboratory and anthropometric measurements from a large number of well children and so adds this important data resource.

## Aims and Objectives

This study aimed to provide age- and sex-adjusted reference values for hsTnT and NT-proBNP obtained from a large cohort of healthy infants, children, and adolescents participating in the Leipzig Research Center for Civilization Diseases (LIFE) Child Study [1]. Secondary aims were to identify associations between these two cardiac markers and with an individual's BMI, pubertal stage, and parameters of lipid metabolism.

## Materials and Methods

2522 Children aged 0.25–18 years (49% female) participating in the prospective longitudinal population-based cohort LIFE Child Study were included. The study, conducted in Leipzig, Germany, consists of a population that is homogeneous, predominantly White Caucasian. Anthropometric data, medical, and medication history together with venous blood samples were obtained from participants at 5057 visits, following standardized protocols. Details about participants, recruitment process, and study protocol have been published previously [1, 12]. Visits were scheduled at approximately 3, 6, and 12 months of age and annually thereafter. The mean difference between the 3- and the 6-month visit was 0.24 (0.04), between the 6- and 12-month visit 0.51, (0.06), and 1.03 (0.1) thereafter. The final sample consisted of 1042 children with 1 visit, 665 children with 2 visits, 439 children with 3 visits, and 278 children with 4+ visits.

Children with known cardiac disease were excluded from the study (*n* = 23, 88 blood samples) as well as children who took cardiovascular medications including digoxin, antihypertensives (Captopril, Losartan, Ramipril, Amlodipine, Enalapril), or anticoagulants (Aspirin, Clopidogrel, Enoxaparin, Apixaban, Certoparin) (*n* = 10, 22 blood samples). 13 Outliers (> 99.75th age- and sex-adjusted percentile) were also excluded from analysis.

Pubertal stages were determined through clinical examination according to Tanner stages [13, 14], and children under 6 years of age were excluded from the subset analyses examining the influence of puberty on hsTnT and NT-proBNP levels.

The LIFE Child Study was designed in conformity with the Declaration of Helsinki and its later amendments [15]. It is registered with ClinicalTrial.gov (NCT02550236) and the study protocol was approved by the Ethical Committee of the University of Leipzig (Reg. No. 264-10-19042010). Written informed consent was obtained from all parents and children over the age of 12 [1].

### Laboratory Values

Blood withdrawal was performed according to study protocol [12]. Serum hsTnT and NT-proBNP concentrations were measured by the Institute of Laboratory Medicine, Clinical Chemistry and Molecular Diagnostics of the University of Leipzig, in accordance with manufacturer’s protocol on an automated laboratory analyzer Cobas 8000 e602 (Roche Diagnostics, Mannheim, Germany), with an electrochemiluminescence immunoassay (ECLIA) based on sandwich principle (Roche Diagnostics, Mannheim, Germany). Primary measuring range for NT-proBNP assay was 5–35,000 ng/L. Primary measuring range for hsTnT assay was 5–10,000 ng/L until conversion to Troponin hs-STAT-Test on Cobas 8000 e801 in 2018 where primary measuring range for Troponin hs-STAT-Test was 3–10,000 ng/L. Comparison measurements were performed between hsTnT and Troponin hs-STAT-Test with very good accordance.

Serum lipid values including cholesterol, triglycerides, as well as HDL- and LDL cholesterol were measured using test kits by Roche Diagnostics GmbH on a ‘Cobas 8000 Clinical Chemistry Analyzer’ with the technique described by Dathan-Stumpf et al. who also provided standard deviation scores (SDS) for these markers [16].

Pre-existing reference values from our laboratory for NT-proBNP were based on levels reported by Albers et al. [4] who utilized varying cut-off values based on age, acquired from a small cohort of 408 patients. For the hsTnT test only the 99th percentile for the adult population was reported as the upper reference value of 14 ng/L (pg/mL) with 10% coefficient variation (CV) precision of 13 ng/L.

### Statistical Analysis

Descriptive statistics are presented as count (%) for categorical variables and mean (standard deviations) for continuous variables (Table 1). To estimate age- and sex-adjusted reference values, obtained values below the detection limits (NT-proBNP < 5 ng/L; 58 out of 4648 below the detection limit, 2017: hsTnT < 3 ng/L; 215 out of 366 below the detection limit and after assay conversion in 2018 to Troponin hs-STAT-Test < 5 ng/L; 2002 out of 3971 below the detection limit) were reported as the lower limit of detection and marked as left-censored.

**Table 1:** Population characteristics and laboratory values on the baseline visit (mean and standard deviations) and distribution of measurements (number and percentage) per pubertal stage for girls and boys

	Male *N* = 1249	Female *N* = 1185
Population characteristics baseline visit		
Age (years)	7.63 (4.93)	8.22 (5.12)
BMI SDS	− 0.03 (0.94)	0.00 (1.00)
Cholesterol SDS	0.02 (1.02)	0.03 (1.00)
HDL SDS	0.10 (1.00)	0.11 (0.95)
LDL SDS	− 0.04 (1.01)	− 0.02 (1.00)
Triglycerides SDS	− 0.11 (0.93)	− 0.09 (0.94)

Distribution of measurements per pubertal stage		
	Male *N* = 1115	Female *N* = 1471
Tanner stage		
1	594 (53.3%)	510 (34.7%)
2	245 (22.0%)	235 (16.0%)
3	72 (6.46%)	199 (13.5%)
4	115 (10.3%)	223 (15.2%)
5	89 (7.98%)	304 (20.7%)

To estimate reference ranges, generalized additive models for location, shape, and scale were applied. This semi-parametric method facilitates estimating reference ranges dependent continuously on age and does not require the subdivision into age intervals. Modeling the reference ranges continuously on age reflects natural development better than artificial partitioning into age intervals. According to the World Health Organization, this method is the best to assess the meaning of measurement values in the context of growth and development [17]. Moreover, it allows transformation of raw measurement values into age- and sex-adjusted SDSs, where 0 represents the expected value and ± 1 indicates a value 1 standard deviation above/below the expected value. The method also allows for left-, right-, and interval-censored data. It is therefore possible to include values below the detection limit into the analyses (as left-censored data points) without the need for imputations. Hence, we assumed a censored Box–Cox-t distribution (BCT) and estimated the distribution parameters for location, scale, skewness, and kurtosis dependent continuously on age and stratified by sex, using GAMLSS [18, 19]. Weights were added to the model to account for multiple measurements per subject. Subsequently, the raw measurement values were transformed to SDS and, in cases of values below the detection limit, also marked as left-censored. Transformations to SDS values were also carried out for BMI and serum lipid values [16, 20].

Associations between the cardiac markers and BMI SDS and SDS of the serum lipids were modeled using GAMLSS assuming a left-censored normal distribution for the outcome variables [19]. To assess associations with pubertal stage and sex, non-transformed values were used as dependent variables. The subject was added as a random effect to account for multiple measurements per child. All statistical analyses were done using R, version 4.0 [21]. The significance level was set to *α* = 0.05.

## Results

### NT-proBNP

NT-proBNP levels were measured in 4648 blood samples, 58 (1.25%) of which were below the detection limit of 5 ng/L.

#### Age and Sex Correlation

**Fig. 1:**
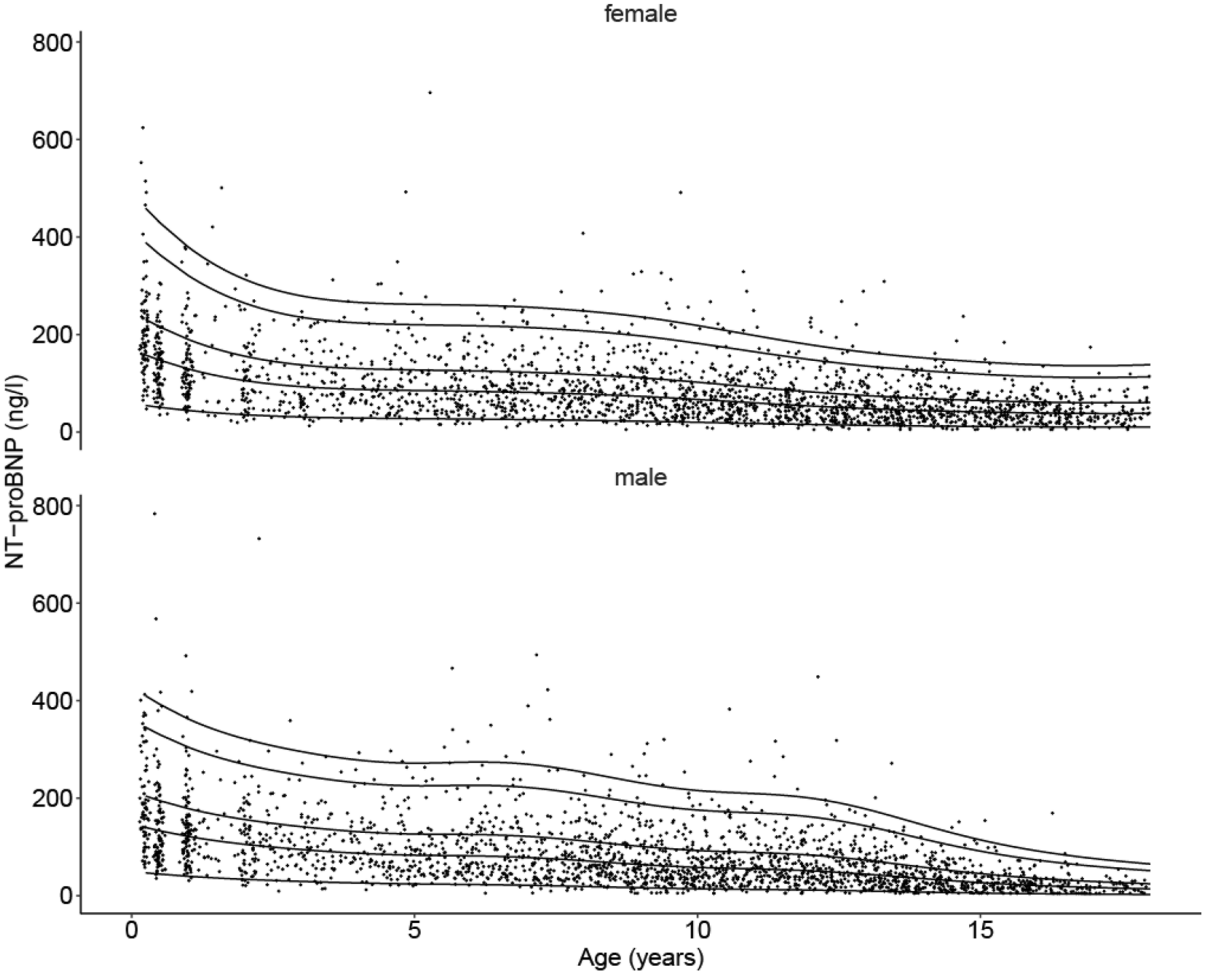
New NT-proBNP percentiles in the pediatric population from the LIFE Child Study cohort (*n* = 2489). 5th, 50th (median), 75th, 95th, and 97.5th (cut-off) percentiles for females and males showing a significant age-dependent decline and generally higher values in females. NT-proBNP levels were determined with ECLIA, Cobas Roche (*n* = 4648)

The estimated 5th, 50th, 75th, 95th, and 97.5th sex-adjusted percentiles are shown in Fig. 1 and Table 2 which also provides a comparison to the previous mentioned percentiles published by Albers et al. [4]. We demonstrate that NT-proBNP values decreased steadily during childhood with generally higher medians in girls than in boys. However, the 97.5th percentiles were only higher in girls for ages ≤ 1 year, 9, 10, and14 years and above.

**Table 2:** Estimated 5th, 50th, 75th, 95th, and 97.5th sex-adjusted percentiles for NT-proBNP (values in ng/L) from the LIFE Child Study cohort (children aged 0.25–18 years) in comparison to the non-sex-specific 75th and 97.5th percentiles from Albers et al. [4]

Life data NT-proBNP (ng/L) select percentiles													Percentiles by Albers et al. [4]			
Age (years)	*n*		5th		50th		75th		95th		**97.5th**		Age (years)	*n*	75th	97.5th
			Male	Female	Male	Female	Male	Female	Male	Female	Male	Female				
0.25	77	77	54.3	46.7	157.8	139.7	230.8	204.1	388.5	345.2	**458.8**	**409.9**				
0.5	92	98	50.6	44.1	147.2	133.0	215.6	194.7	363.5	330.3	**429.4**	**392.4**				
1	92	121	44.1	39.8	129.4	121.5	190.0	178.6	321.2	304.6	**379.7**	**362.6**	1–3	13	231.2	**319.9**
2	81	98	35.5	33.5	105.5	105.3	155.5	156.2	264.4	269.2	**313.1**	**321.5**				
3	75	82	30.8	29.2	93.0	94.6	137.7	141.5	235.4	246.7	**279.3**	**295.6**				
4	79	77	28.6	26.0	87.5	87.0	130.2	131.3	223.8	231.4	**265.9**	**278.3**	4–6	21	112.6	**189.7**
5	98	104	27.4	24.0	85.2	82.9	127.3	126.3	220.1	225.3	**262.0**	**271.8**				
6	113	115	26.5	22.9	83.5	81.6	125.4	125.4	218.1	226.2	**260.1**	**273.9**				
7	128	130	25.4	21.2	81.3	78.3	122.6	121.5	214.5	221.9	**256.2**	**269.6**	7–9	32	94.4	**144.7**
8	136	162	23.9	18.7	77.8	71.6	117.8	112.2	207.4	207.3	**248.1**	**252.8**				
9	146	178	22.2	16.1	73.1	63.8	111.3	101.0	197.0	189.0	**236.2**	**231.3**				
10	155	174	19.9	14.1	66.8	57.9	102.1	92.7	181.9	175.6	**218.4**	**215.7**	10	11	72.5	**112.4**
11	154	161	17.6	12.8	59.8	54.8	91.9	88.5	164.7	169.8	**198.1**	**209.4**	11	69	93.4	**317.1**
12	158	174	15.4	11.5	53.3	50.9	82.3	83.1	148.4	161.6	**178.9**	**200.1**	12	21	95.0	**186.4**
13	163	160	13.6	9.5	48.0	43.8	74.6	72.3	135.2	142.4	**163.3**	**177.0**	13	23	113.6	**369.9**
14	162	168	12.3	7.2	44.1	34.8	68.9	58.1	125.7	115.9	**152.0**	**144.6**	14	18	68.2	**362.8**
15	130	117	11.3	5.3	41.2	26.7	64.6	45.1	118.6	91.3	**143.7**	**114.4**	15	24	73.6	**216.7**
16	127	100	10.5	4.0	39.0	20.8	61.4	35.6	113.5	73.0	**137.8**	**91.8**	16	24	84.9	**206.0**
17	78	50	10.1	3.1	37.9	16.8	60.1	29.0	111.7	60.3	**135.9**	**76.2**	17	24	71	**134.9**

97.5th percentiles are highlighted in bold

#### BMI

After adjusting for age, there was a significant association between increasing BMI SDS and lower NT-proBNP levels in boys (*β* =  − 0.12, *p* < 0.0001) and in girls (*β* =  − 0.11, *p* < 0.0001), with no evidence for a difference in effect between the sexes (*β*_interaction_ =  − 0.001, *p* = 0.56) and an overall effect of *β* =  − 0.11, *p* < 0.0001.

#### Metabolic Markers

After adjusting for age, we identified statistically significant negative associations between SDS of lipid levels (cholesterol, HDL cholesterol, LDL cholesterol, and triglycerides) and SDS of NT-proBNP levels. In general, the association between serum lipid levels and NT-proBNP levels were more pronounced in males than in females. This difference in effects reached statistical significance for cholesterol, LDL cholesterol, and triglycerides [cholesterol: *β*_boys_ =  − 0.11, *p* < 0.0001; *β*_girls_ =  − 0.06, *p* < 0.0001, *β*_interaction_ = 0.05, *p* = 0.018; LDL: *β*_boys_ =  − 0.08, *p* < 0.0001; *β*_girls_ =  − 0.03, *p* = 0.014; *β*_interaction_ = 0.05, *p* = 0.014; triglycerides: *β*_boys_ =  − 0.16, *p* < 0.0001; *β*_girls_ =  − 0.11, *p* < 0.0001, *β*_interaction_ = 0.05, *p* = 0.01]. There was no significant interaction found for HDL cholesterol (*β*_interaction_ = 0.020, *p* = 0.35) and the overall effect was *β* =  − 0.04, *p* = 0.0001.

#### Puberty

**Fig. 2:**
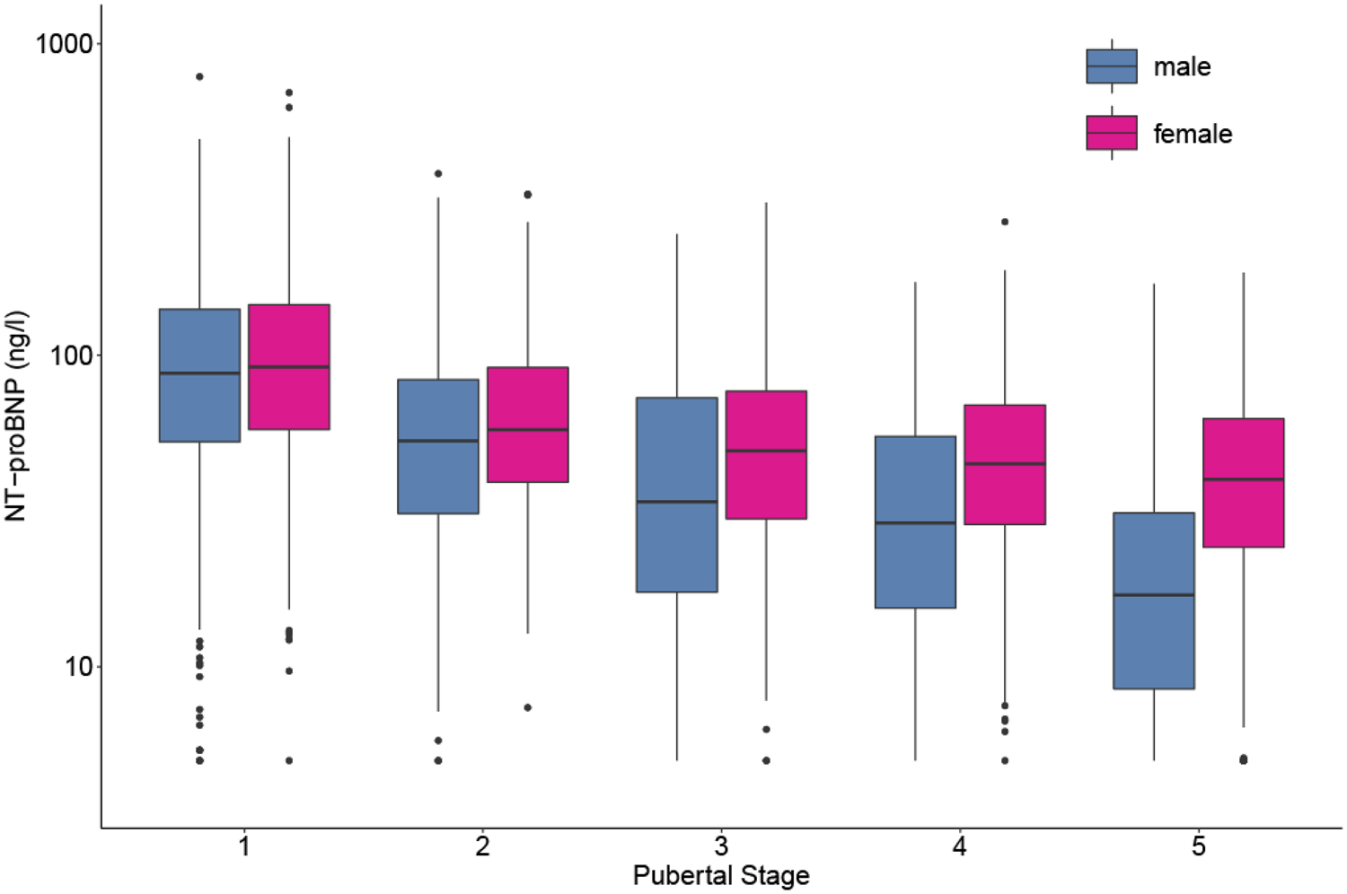
Influence of pubertal stage on NT-proBNP levels in the LIFE Child Study cohort. Box-plots represent the 25th and 75th percentiles (boxes) and 50th percentile (mid line). NT-proBNP levels were higher in girls than in boys with an increasing effect in advancing puberty and dropped significantly in both genders again with an increasing effect in ongoing puberty until stage 4, from where only levels in males decreased further. NT-proBNP levels were determined with ECLIA, Cobas Roche (*n* = 2537)

NT-proBNP levels fell in both sexes through pubertal stages 1 to 4 with decreasing effect sizes for each step in pubertal stage (Fig. 2). The decline in value from pubertal stages 1 to 2 in boys (*β* =  − 33.4, *p* < 0.0001) and girls (*β* =  − 33.0, *p* < 0.0001) was greater than the subsequent fall in values [stages 2–3 (*β*_boys_ =  − 19.7, *p* = 0.0009; *β*_girls_ =  − 16.2, *p* = 0.0001), stages 3–4 (*β*_boys_ =  − 12.6, *p* = 0.057; *β*_girls_ =  − 9.7, *p* = 0.042)]. The downward trend continued between stages 4 and 5 but was only of statistical significance in boys (*β*_boys_ =  − 16.1, *p* = 0.009; *β*_girls_ =  − 6.5, *p* = 0.066)].

Girls showed consistently higher NT-proBNP levels than boys in all pubertal stages, which became more apparent later in puberty as the fall in values with progressing puberty was less marked than in their male counterparts. This was statistically significant for pubertal stages 1 (*β* = 6.7, *p* = 0.018), 4 (*β* = 14.5, *p* = 0.004), and 5 (*β* = 23.6, *p* < 0.0001), but not for pubertal stages 2 (*β* = 7.1, *p* = 0.076) or 3 (*β* = 10.6, *p* = 0.080).

### hs-Troponin T

In our cohort, 50.36% of the 4337 measured hsTnT levels were below the lower limit of detection of the assay (5 ng/L from 2018 or 3 ng/L prior to 2018).

#### Age and Sex Correlation

**Fig. 3:**
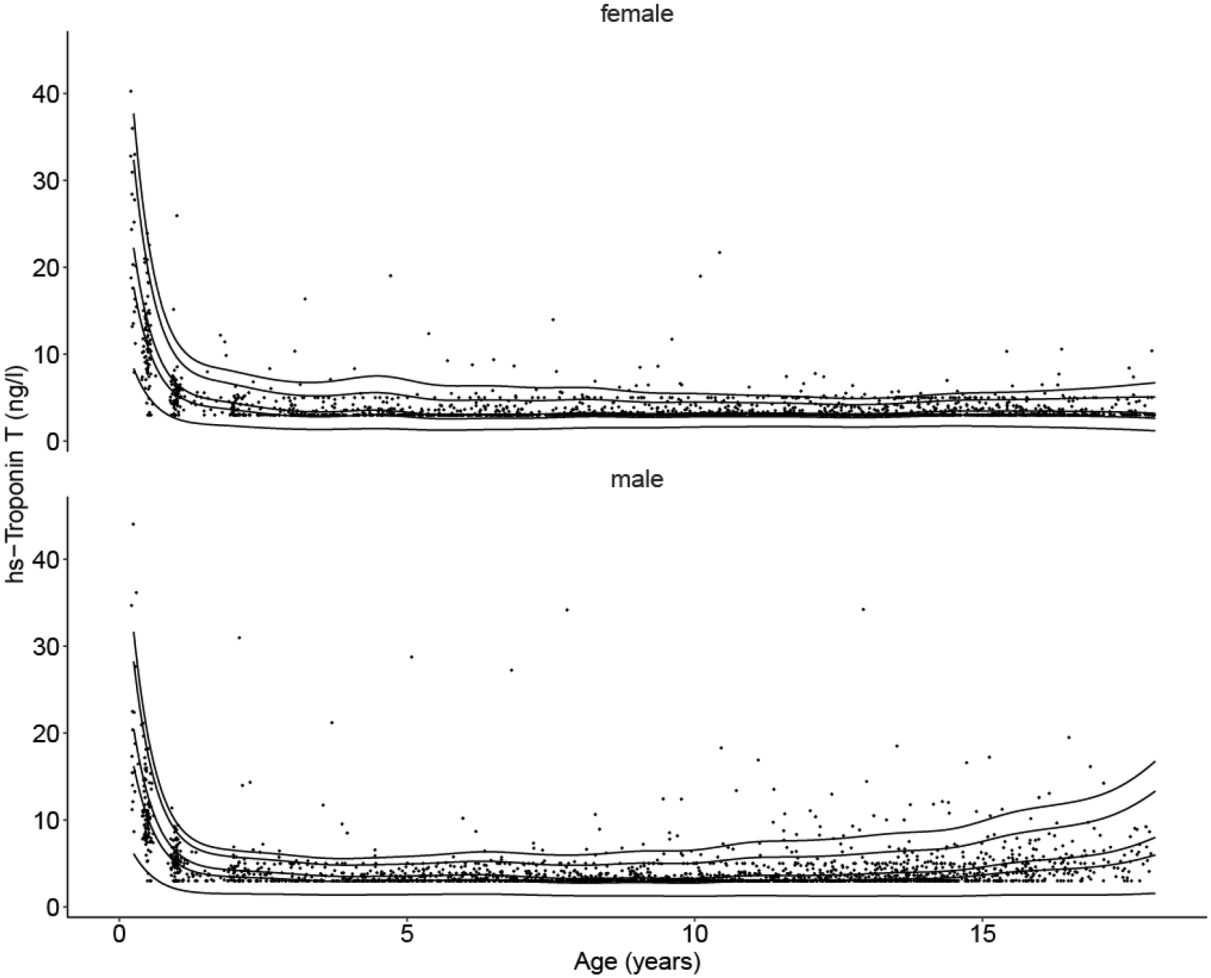
New hs-Troponin T percentiles in the pediatric population from the LIFE Child Study cohort (*n* = 2489). 5th, 50th (median), 75th, 95th, and 97.5th (cut-off) percentiles for females and males showing a significant age-dependent decline in the first year of life and generally values below the adult cut-off, as well as a surprising rise in teenage boys. hs-Troponin T levels were determined with ECLIA, Cobas Roche (*n* = 4337)

The estimated 5th, 50th, 75th, 95th, and 97.5th sex-adjusted percentiles are shown in Table 3 and Fig. 3. hsTnT levels in our cohort for the 97.5th percentile were found to be above the adult cut-off limit of 14 ng/L in children up to the age of 6 months and again in older males between 17.5 and 18 years of age. Under the age of 2 years, females tended to have higher hsTnT levels than their male counterparts.

**Table 3:** Estimated 5th, 50th, 75th, 95th, and 97.5th sex-adjusted percentiles for hs-Troponin T (values in ng/L) from the LIFE Child Study cohort (children aged 0.25–18 years)

Life data hs-Troponin-T (ng/L) select percentiles												
Age (years)	*n*		5th		50th		75th		95th		97.5th	
	Male	Female	Male	Female	Male	Female	Male	Female	Male	Female	Male	Female
0.25	20	17	8.35	6.11	17.71	16.22	22.25	20.47	32.38	28.26	37.72	31.66
0.5	79	89	5.14	3.92	10.81	10.12	13.51	12.71	19.67	17.49	23.04	19.61
1	88	107	2.56	2.05	5.32	5.04	6.58	6.26	9.63	8.58	11.43	9.64
2	77	83	1.73	1.50	3.59	3.45	4.37	4.21	6.52	5.75	8.02	6.50
3	74	76	1.39	1.44	2.90	3.15	3.49	3.80	5.33	5.19	6.83	5.93
4	74	73	1.39	1.37	2.91	2.93	3.47	3.50	5.41	4.81	7.18	5.56
5	93	102	1.36	1.42	2.81	2.99	3.31	3.55	5.20	4.94	7.05	5.80
6	111	111	1.28	1.48	2.58	3.12	3.02	3.69	4.70	5.21	6.36	6.24
7	118	127	1.36	1.40	2.64	2.96	3.08	3.49	4.67	5.04	6.20	6.16
8	130	155	1.51	1.28	2.81	2.75	3.25	3.24	4.79	4.78	6.18	5.98
9	142	173	1.53	1.28	2.74	2.81	3.16	3.33	4.52	5.02	5.65	6.41
10	149	174	1.62	1.22	2.80	2.74	3.22	3.26	4.49	5.04	5.47	6.54
11	152	155	1.66	1.30	2.81	3.01	3.23	3.60	4.42	5.66	5.27	7.45
12	157	172	1.66	1.25	2.77	3.00	3.19	3.61	4.31	5.78	5.07	7.67
13	158	154	1.62	1.25	2.71	3.12	3.13	3.80	4.21	6.16	4.91	8.18
14	156	168	1.71	1.23	2.88	3.25	3.34	4.01	4.51	6.55	5.26	8.66
15	123	105	1.72	1.30	2.97	3.67	3.47	4.59	4.75	7.54	5.57	9.88
16	122	94	1.61	1.38	2.93	4.22	3.45	5.38	4.83	8.87	5.77	11.48
17	74	48	1.45	1.37	2.84	4.64	3.39	6.04	4.97	10.00	6.14	12.77
18	36	21	1.18	1.56	2.63	5.98	3.18	8.00	5.07	13.31	6.71	16.76

#### BMI

After adjusting for age, we found a significantly positive association between BMI SDS and hsTnT SDS in boys (*β* = 0.04, *p* = 0.030) but not in girls (*β* =  − 0.010, *p* = 0.63).

#### Metabolic Markers

Similar to our findings for NT-proBNP, there was an association observed between lipid levels and hsTnT which was more apparent in males than in females. There was a marginally significant negative association between hsTnT SDS and LDL SDS in boys (*β* =  − 0.04, *p* = 0.046) but not in girls (*β* = 0.007, *p* = 0.73) and a significantly negative association between hsTnT SDS and triglycerides SDS in boys (*β* =  − 0.044, *p* = 0.018) but not girls (*β* =  − 0.030, *p* = 0.14). There was no meaningful association found between hsTnT SDS and cholesterol SDS or HDL SDS.

#### Puberty

**Fig. 4:**
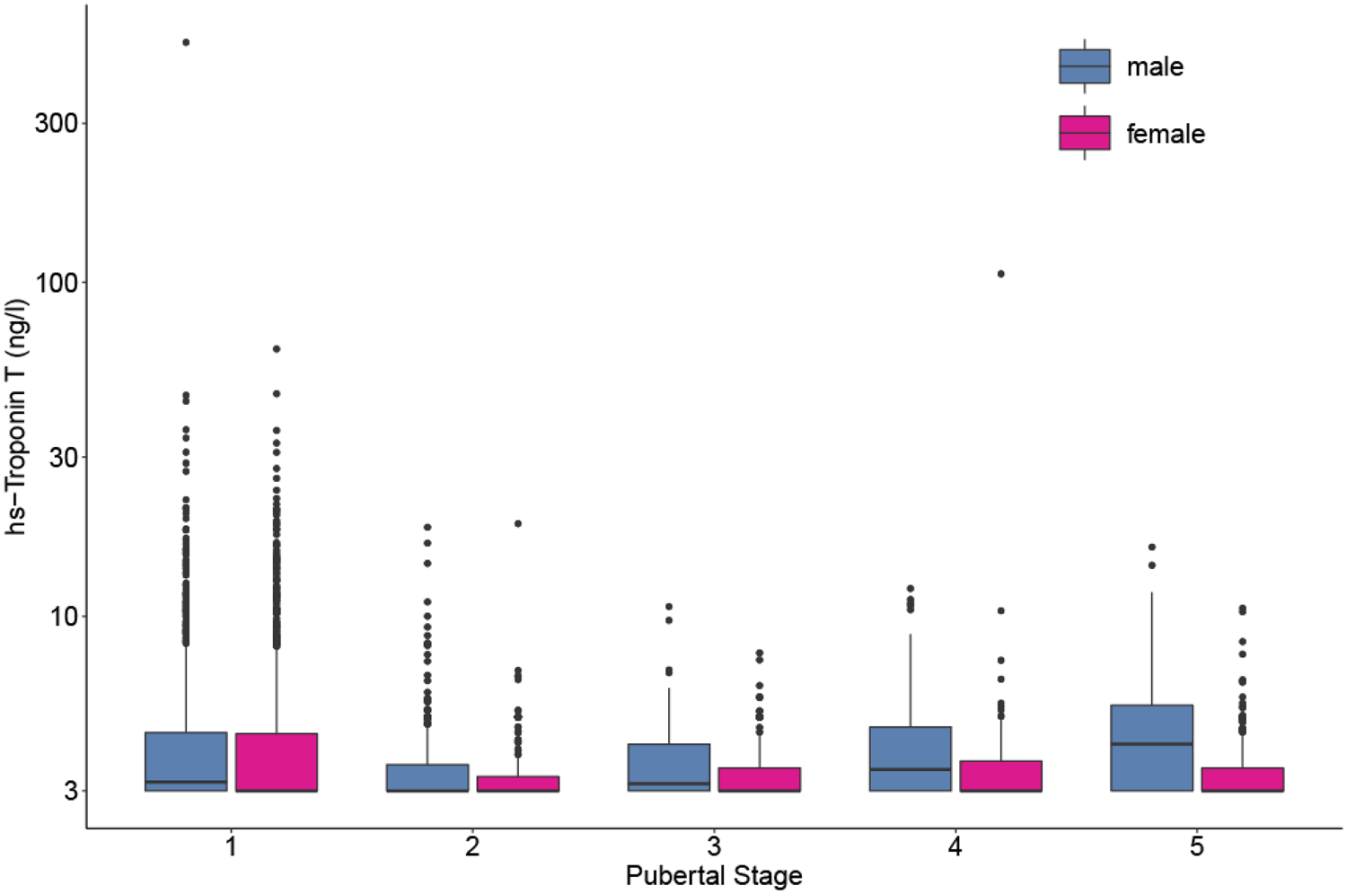
Influence of pubertal stage on hs-Troponin levels in the LIFE Child Study cohort. Box-plots represent the 25th and 75th percentiles (boxes) and 50th percentile (mid line). Hs-Troponin levels were lower in females with an increasing effect during puberty and rose from pubertal stage 2 onward in males. Levels were determined with ECLIA, Cobas Roche (*n* = 2453, levels of children older than 6 years of age)

During all pubertal stages girls had lower hsTnT levels than boys, with increasing effect sizes with increasing pubertal stage (*β*_p1_ =  − 0.19, *p* = 0.39; *β*_p2_ =  − 0.83, *p* = 0.03; *β*_p3_ =  − 0.61, *p* = 0.28; *β*_p4_ =  − 0.99, *p* = 0.05; *β*_p5_ =  − 2.94, *p* < 0.0001). From pubertal stage 1 to 2, the levels dropped significantly in both boys (*β* =  − 1.0, *p* = 0.0007) and girls (*β* =  − 1.7, *p* < 0.0001). We could not find statistically significant differences in levels between subsequent pubertal stages in either sex. In males, we saw an increase in hsTnT levels from pubertal stage 2 onward, reaching statistical significance between pubertal stage 5 and pubertal stage 2 (*β* = 1.9, *p* = 0.0004, Fig. 4).

### hsTnT and NT-proBNP

**Fig. 5:**
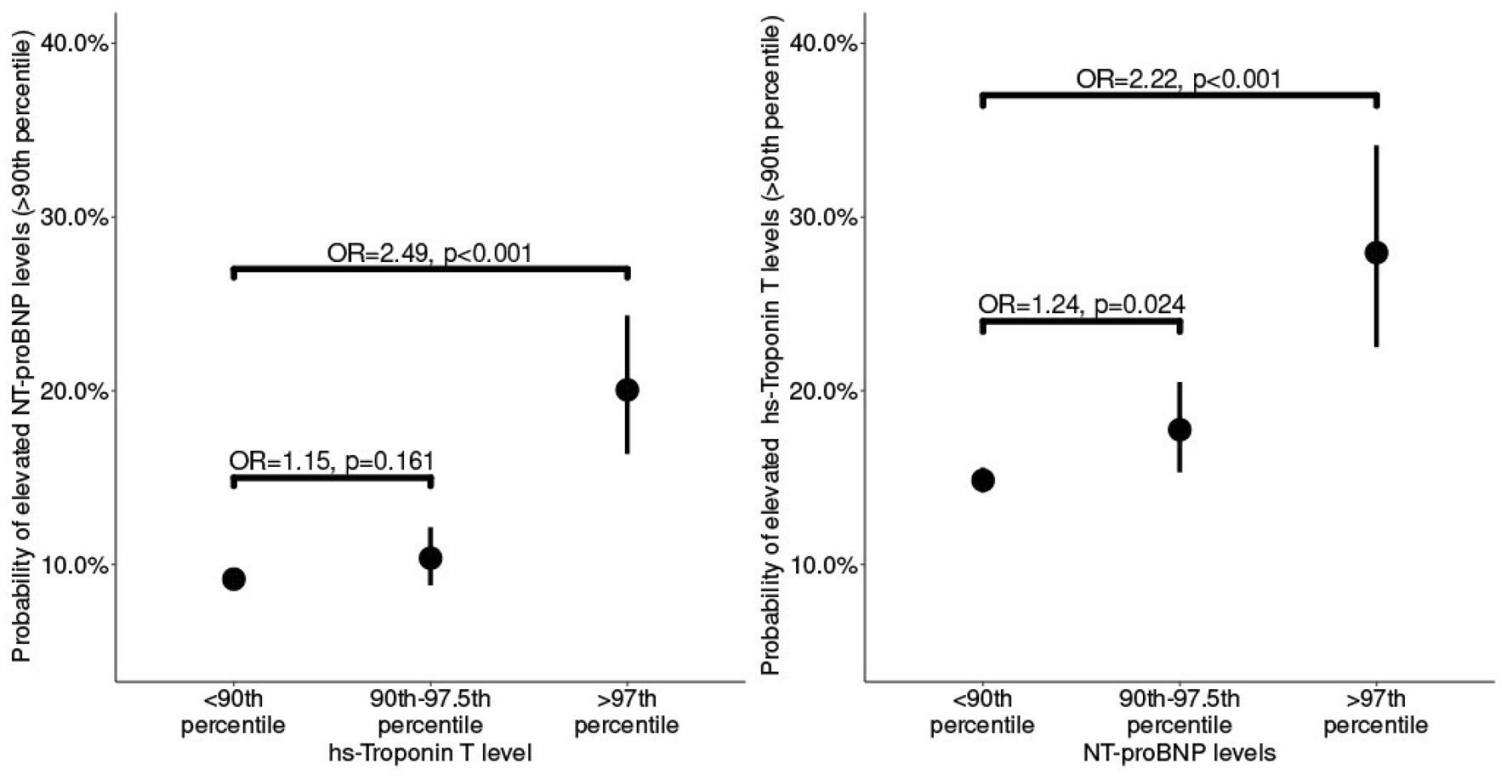
NT-proBNP and hs-Troponin T levels above the 90th percentile increase the probability of the alternate marker to be above the 90th percentile. Our study cohort showed no direct correlation between NT-proBNP and hs-Troponin T values, but an increasing probability of levels above the 90th percentile for NT-proBNP, respectively, hs-Troponin T, when the other marker was above the 97th versus the 90th percentile

While there was no direct correlation between NT-proBNP and hsTnT values in our cohort, the probability of a level above the 90th percentile for NT-proBNP or hsTnT was higher when the other marker was also elevated above the 90th percentile. The chance of higher values was even greater in children with levels above the 97th percentile (probability of elevated NT-proBNP levels: OR 2.49, *p* < 0.001, hsTnT levels: OR 2.22, *p* < 0.001, Fig. 5).

## Discussion

Our study provides the first specific pediatric percentiles for NT-proBNP and hsTnT based on a large sample of healthy children. Additionally, we have evidence that pubertal stage, BMI, and lipid levels impact the levels of these cardiac markers.

### ProBNP

#### Age and Sex Correlation

Several previous small studies have aimed to estimate reference ranges for proBNP (BNP and/or NT-proBNP) based on age and sex [3–6, 22, 23]. In 2014, Cantinotti et al. summarized the previous literature in their review on plasma BNP and NT-proBNP measurements in children and stated that BNP concentrations remain consistent after a rapid decline in the first month of life until the age of 12 years [24]. One of the included studies by Nir et al. pooled data from four different cohorts (*n* = 690 children, aged 0–18 years, 47% males) [3]. In line with our results, they revealed a strong correlation between increasing age and decreasing 97.5th percentile levels but with generally higher cut-off values than those seen in our study. For example, within the age group 1–2 years Nir et al. stated 675 ng/L (*n* = 38) as cut-off compared to 316–379 ng/L (*n* = 392) in our cohort. This was also replicated in the teenage group; Nir et al. stated 207 pg/mL (*n* = 116) as cut-off for children between 14 and 18 years of age, whereas our cut-offs for these ages were below 152.1 ng/L (*n* = 990). The clear downward trend we demonstrate between each age range used by Nir et al. was not illustrated in their age group-based data. Comparing the 50th percentile (Nir et al. = 34 ng/L), we found equivalent values in our teenage cohort (14–44 ng/L across the respective age range). It should be noted that their group sizes of *n* = 38 and 116 are considered too small to generate reliable 97.5th percentiles, whereas our sample sizes for the equivalent ages comprised 392 and 990 measurements.

Soldin et al. also suggested higher 97.5th percentiles, for example, they reported a cut-off of 5012 pg/mL (*n* = 105) for boys between 1 and 3 years and for girls of the same age of 2512 pg/mL (*n* = 59) [22] compared to children of the same age in our cohort who had cut-off values below 400 ng/L (*n* = 559). In this study none of the age groups reached a sample size of 100, instead varying between 26 and 91 for the ages > 3 months, so again generating a reliable 97.5th percentile is not feasible.

The Canadian CALIPER cohort based on 484 healthy subjects (aged 0–18 years) supported our findings for lower cut-off values above 1 year of age but suggested a very high cut-off for children below 1 year (99th percentiles for 0–12 months: 5272 ng/L and for 1–18 years: 216 ng/L) [5]. These differences in the cut-offs can be explained by the division into only two broad age groups in the CALIPER study and so, neither the changes in proBNP levels that occur rapidly in the first year of life, nor changes with ongoing puberty are captured in the study.

Data from the Australian LOOK cohort derived from less than 400 healthy children per age group (8-, 10- and 12-year-old) also collaborated our findings of lower cut-offs. Their proposed 97.5th percentiles were even lower than those derived from our cohort with values varying between 160 and 190 ng/L compared to our values between 178.9 and 252.8 ng/L. Again, the median values were similar to other published values between 42.1 and 60.0 ng/L and correlate with our median values of between 50.9 and 77.8 ng/L in these age ranges [6].

Compared to the widely used reference values published by Albers et al. (Table 2), we could demonstrate a significant and steady decrease of NT-proBNP levels during childhood with higher cut-off values for younger children and lower cut-off levels for teenagers. Our clear age-dependent trend relies on a larger sample with a much lower level of uncertainty for the 97.5th percentiles compared to the small age groups of about 25 children in the study by Albers et al. [4]. Considering these facts, the estimates concurred reasonably well. For example, Albers et al. proposed 320 ng/L for children from 1 to 3 years, whereas we found decreasing values between 380 and 280 ng/L for the same age range in our cohort.

The similar median values provided by Nir et al. and Koerbin et al. to our study suggest that the significant differences in the 97.5th percentiles may be caused by differing compositions of study cohorts both in terms of size (generating possible sampling error) and baseline health characteristics. The principal advantage of our study lies in our significantly larger sample sizes that generate more reliable values.

Regarding differences between the sexes, Nir et al., Koerbin et al., and Lam et al. found no discernible difference in values. Cantinotti et al. found no difference between the sexes up to 10–14 years of age, while Soldin et al. found higher 97.5th percentiles in boys than in girls in almost all age groups, excluding newborns and children between 3 and 10 years old, where girls had higher levels [3, 5, 6, 22, 24]. In our cohort, girls were found to have significantly higher 97.5th percentiles than boys until the age of 1 year and then again above 14 years of age. The higher values in males that Soldin et al. reported for those aged 15–18 years and between 18 and 21 years (*n*_men_ = 61, *n*_women_ = 51) are contradicted both by our data and that obtained from adult studies [7, 22, 25]. For a better understanding of the influence of the sex on proBNP levels in childhood further investigation including correlation with sex hormone levels is required.

#### Adult Data on Age Dependency and Sex Hormones

Several studies in adults have demonstrated higher NT-proBNP values in females and with advancing age. The Framingham Heart Study from 2011 found a doubling of the upper reference limits between 20 and 60 years of age (*n* = 2200). The explanation of age dependency provided implied that androgens might inhibit BNP secretion. This, however, cannot be the full explanation because there was a similar increase in NT-proBNP values seen in both men and women despite larger changes in the androgen levels of men [25]. Nevertheless, this finding is supported by subsequent data from Dallas Heart Study Group (DHS), where NT-proBNP levels of 682 women aged 35 to 49 years were found to correlate positively with SHGB and inversely with free androgen index and calculated free testosterone [26].

#### Puberty

For the pediatric population, associations between proBNP, sex hormones, and puberty are less clear. Koch et al. reported higher BNP values in girls during the second decade of life and a correlation of BNP levels with progression of Tanner stages, but did not identify any difference between pre- and post-pubertal boys [23]. A finding also reported by Saenger et al. [27].

In contrast to these two studies, which combined less than 1000 children and who had concurrent minor illnesses/surgeries at the time of sample collection, our data of more than double the number of well children clearly demonstrated a significant decrease in NT-proBNP levels for both sexes with ongoing puberty; in girls until stage 4, in boys until post-puberty. We also found, in accordance with adult data, consistently higher NT-proBNP levels across all pubertal stages in girls, with increasing effect size during advancing puberty.

#### Obesity and Metabolic Syndrome

Sutahar et al. demonstrated in their cohort of more than 8000 adults an inverse association between NT-proBNP and BMI [7]. Small sample numbers limit equipoise in prior pediatric studies. Pervanidou et al. collected data from 96 children aged 10–16 years, of whom approximately half were obese. They analyzed the potential link between NT-proBNP and adiponectin levels, which are known to be lower in subjects with obesity. A significant correlation was detected in girls where lower adiponectin levels were associated with lower NT-proBNP levels, with an increasing effect seen with rising BMI [28]. Among boys, this was reversed, where leaner boys were found to have lower NT-proBNP concentrations than their obese counterparts [29]. Siervo et al. in their study of 151 children (*n* = 38 with BMI *z*-score ≥ 1.64), however, found no association between BMI *z*-score and hsTnT or NT-proBNP levels [10]. A finding replicated by Battal et al., who evaluated 68 obese and 38 lean matched-controls and found no significant difference in NT-proBNP levels, nor any difference between obese children with or without metabolic syndrome, hypertension or insulin resistance, and NT-proBNP levels [11].

Our data concur with the results reported in the adult literature clearly demonstrating (after adjustment for age and sex) a negative association between BMI SDS and NT-proBNP levels [7–9]. We also observed significant negative associations between SDS of lipid levels (cholesterol, LDL cholesterol, and triglycerides) and NT-proBNP levels, which was more pronounced in boys.

### Troponin

#### Age and Sex Correlation

Several small studies suggest higher Troponin T and I values in neonates and young infants that then decline to adult-like values in older children and teenagers [30–33].

Caselli et al. investigated Troponin I values in 375 healthy children, including 36 neonates. They estimated 95th percentiles for newborns (139 ng/L), infants (85 ng/L), children 1–10 years old (39 ng/L), and children 10–18 years old (6 ng/L). While the 95th percentile for the age range 10–18 years corresponds well with our 95th percentile for hsTnT (4–5 ng/L for females and 5–13 ng/L for males), their estimates for infants aged between 1 month and 1 year and children between 1 and 10 years were markedly higher than ours. The sample sizes in Caselli et al. were again too small to accurately estimate a 95th percentile (*n* = 57 and 65, respectively) relying mostly on two outliers. Interestingly, in their multivariate analysis only age was found to be an independent predictor, sex was not [31].

Franzini et al. found significantly lower 99th percentiles for Troponin T in those aged 10–20 years with a progressive rise in values for those aged 20–64 to those over 65 years of age. Here, in all age groups, males showed higher levels than females [32].

The 99th percentiles for hsTnT estimated from the CALIPER cohort based on serum samples from 245 healthy subjects created age distributions based on their measurement values of < 6 months, 6–12 months, and 1–18 years with cut-off values of 87, 39, and 11 ng/L, respectively. Their analysis confirmed that the highest concentrations are present in the youngest age group but they could not identify sex dependency in Troponin levels [5]. The reference limits offered for the first six months of life were not comparable with our study as we did not have data for children less than three months of age. For the age interval 0.5–1 year, we found considerably lower 97.5th percentiles.

In summary, compared to the aforementioned studies, the 97.5th percentiles for hsTnT in our cohort were lower. The differences can be explained by statistical power generated from larger sample sizes, as well as differences in composition of the study population approach in handling the age dependency, methods of quantile estimation, and in laboratory methods.

#### Puberty

Although no prior study has investigated the effect of puberty on Troponin levels, studies by Cirer-Sastre et al. and Legaz-Arrese et al. have assessed the effect of exercise-induced release of cardiac markers at different stages of puberty [34, 35]. We could demonstrate that hsTnT levels in boys rise from pubertal stage 2 onward and that in all pubertal stages, girls have consistently lower levels than boys, with an increasing effect during puberty.

#### Obesity and Metabolic Syndrome

Pervanidou et al. compared a small group of obese children with and without metabolic syndrome to non-obese children, observing significantly higher but still normal Troponin levels in the children with metabolic syndrome. They suggest that it is not obesity alone but obesity-induced metabolic changes that are linked to increased Troponin levels [36]. We could demonstrate that higher lipid levels are not linked to higher hsTnT levels and, in contrast, LDL SDS or triglycerides SDS are reversely correlated to hsTnT SDS in boys, despite the positive association between BMI SDS and hsTnT SDS.

### Study Strengths and Weaknesses

Our strengths lie in being the largest pediatric cohort study to date, facilitating the estimation of sex- and age-related reference limits for cardiac markers in this population. Our statistical analysis is in accordance with that recommended by the World Health Organization, using a continuous approach to estimate the age dependency as the optimal method to assess the meaning of measurement values in the context of sex and age [17, 37].

The main weakness of our study is the absence of data from newborns and infants younger than 3 months, especially as within this age group, they seem to have initially high and then rapid declining values for both cardiac markers. Further studies are needed, especially in the first year of life, to complement our data. In one of our smaller cohort groups, males aged > 17 years, we found higher than adult values for hsTnT, which require further investigation and may be due to an inadequate sample size.

Adult data have demonstrated the impact of race and ethnicity on proBNP levels where Black and Chinese individuals had significantly lower levels than their White counterparts suggesting a genetic influence [38, 39]. This has not been investigated in pediatric cohorts, unfortunately, we were not able to look for this interesting point as our study cohort is based in East Germany with a homogeneous, predominantly White Caucasian population and so is inadequately powered to demonstrate any differences between races or ethnicities.

## Conclusion

Our study provides reliable age-dependent reference values for NT-proBNP and hsTnT for each sex, obtained from a large, healthy pediatric cohort. Our data question a number of previous published findings. We have demonstrated remarkably higher cut-off values for NT-proBNP in pre-pubertal children than the hitherto existing reference values, as well as lower cut-offs in children aged 11 and older. Furthermore, we have demonstrated a steady and consistent decline of the medians and the cut-off values with age. For hsTnT levels in childhood, our study confirms peaks above the adult cut-off limit of 14 ng/L in infants (3 months and 6 months of age), with girls having slightly higher levels. Boys showed a steady upward trend with increasing pubertal state and a positive correlation with weight status. We also found associations with serum lipid levels with a greater effect in boys. Further investigation is warranted to understand the inter-current relationship between cardiac markers and metabolic factors, including BMI, pubertal stages, and lipid levels.

We suggest a revision, verification, and adjustment of cut-off values for both of these cardiac markers for use in pediatric clinical practice.

## Abbreviations

BMI: Body mass index
BNP: Brain natriuretic peptide
CV: Coefficient variation
COVID-19: Coronavirus disease 2019
DHS: Dallas Heart Study
ECLIA: Electrochemiluminescence immunoassay
HDL cholesterol: High-density lipoprotein cholesterol
hsTnT: High-sensitive Troponin T
LDL cholesterol: Low-density lipoprotein cholesterol
LIFE Child: Leipzig Research Center for Civilization Diseases (LIFE) Child
NT-proBNP: N-terminal-pro hormone brain natriuretic peptide
proBNP: Pro hormone brain natriuretic peptide
SDS: Standard deviation score
SHGB: Sex hormone-binding globulin
